# Mitochondrial Protection and Against Glutamate Neurotoxicity via Shh/Ptch1 Signaling Pathway to Ameliorate Cognitive Dysfunction by Kaixin San in Multi-Infarct Dementia Rats

**DOI:** 10.1155/2021/5590745

**Published:** 2021-07-09

**Authors:** Xiaoqiong Li, Wen Wen, Ping Li, Ying Fu, Hao Chen, Fushun Wang, Yuan Dai, Shijun Xu

**Affiliations:** ^1^Institute of Material Medica Integration and Transformation for Brain Disorders, Chengdu University of Traditional Chinese Medicine, Chengdu, Sichuan 611137, China; ^2^School of Pharmacy, Chengdu University of Traditional Chinese Medicine, Chengdu, Sichuan 611137, China; ^3^Institute of Brain and Psychological Science, Sichuan Normal University, Chengdu 610060, China; ^4^School of Health Preservation and Rehabilitation, Chengdu University of Traditional Chinese Medicine, Chengdu, Sichuan 610075, China; ^5^State Key Laboratory of Southwestern Chinese Medicine Resources, Chengdu, Sichuan 611137, China

## Abstract

Multi-infarct dementia (MID), a prominent subtype of vascular dementia (VD), is responsible for at least 15 to 20 percent of dementia in the elderly. Mitochondrial dysfunctions and glutamate neurotoxicity due to chronic hypoperfusion and oxidative stress were regarded as the major risk factors in the pathogenesis. *Kaixin San* (KXS), a classic prescription of *Beiji Qianjin Yaofang*, was applied to treatment for “amnesia” and has been demonstrated to alleviate the cognitive deficit in a variety of dementias, including MID. However, little is known whether mitochondria and glutamate are associated with the protection of KXS in MID treatment. The aim of this study was to investigate the role of KXS in improving the cognitive function of MID rats through strengthening mitochondrial functions and antagonizing glutamate neurotoxicity via the Shh/Ptch1 signaling pathway. Our data showed that KXS significantly ameliorated memory impairment and hippocampal neuron damage in MID rats. Moreover, KXS improved hippocampal mitochondrial functions by reducing the degree of mitochondrial swelling, increasing the mitochondrial membrane potential (MMP), and elevating the energy charge (EC) and ATP content in MID rats. As expected, the concentration of glutamate and the expression of p-NMDAR1 were significantly reduced by KXS in the brain tissue of MID rats. Furthermore, our results showed that KXS noticeably activated the Shh/Ptch1 signaling pathway which was demonstrated by remarkable elevations of Ptch1, Smo, and Gli1 protein levels in the brain tissue of MID rats. Intriguingly, the inhibition of the Shh signaling pathway with cyclopamine significantly inhibited the protective effects of KXS on glutamate-induced neurotoxicity in PC12 cells. To sum up, these findings suggested that KXS protected MID rats from memory loss by rescuing mitochondrial functions as well as against glutamate neurotoxicity through activating Shh/Ptch1 signaling pathway.

## 1. Introduction

Multi-infarct dementia (MID) is a prominent subtype of vascular dementia (VD) and is responsible for at least 15 to 20 percent of dementia in the elderly. It is widely known that multiple lesions, infarction of small arteries in the cerebral gray-white matter, and neuronal degeneration are its characteristic lesion [1, 2]. MID is resulted from atherosclerotic disease in large arteries (e.g., carotid) with widespread thromboembolic events, accompanied with chronic cerebral hypoperfusion, which leads to obvious brain pathological damage and cognitive impairment [3–5]. Cerebral ischemia caused by chronic hypoperfusion could induce cerebral hypoxic and result in the production of reactive oxygen species (ROS) and excitatory amino acid (EAA), such as glutamate [6], which directly impair mitochondrial homeostasis and energy production [4, 7]. Therefore, flawed energy metabolism and accumulated EAA in the brain are usually considered as the hallmarks in the pathogenesis of MID. The mitochondria are a cell's power packs and responsible for the energy supply of nerve cells as well as neurotoxicity through oxidative phosphorylation. The hippocampal structure in the brain is the core division for spatial cognitive activity, and its mitochondria are very sensitive to ROS and energy deficit, thus, hippocampal mitochondrial dysfunction plays a key role in cerebral ischemia-induced cognitive impairment [8]. In addition, glutamate outflow and excessive extracellular glutamate accumulation resulted from the death of nerve cells due to energy deficit in turn could also lead to ischemic neuronal death and cognitive disorder [9, 10]. Previous studies suggested that cognitive deficit could be alleviated by reversing brain mitochondrial dysfunction and reducing excessive glutamate content in MID rats [4, 11, 12].

The sonic hedgehog (Shh)/patched1 (Ptch1) pathway plays an important role in cell development, proliferation, pattern, and fate specification [13–15], which mainly includes Shh ligand, its receptor patched (Ptch1), and the pathway activator smoothened (Smo) receptors. Briefly, the binding of Shh to Ptch1 results in release of Smo, Smo activation, and subsequent activation of Gli transcription factors (Gli1, Gli2, and Gli3) [16]. Current investigations had suggested that activating Shh/Ptch1 signaling pathway could improve neurological function and alleviate vascular cognitive impairment [17–19] and protect brain tissue from cerebral ischemia injury [20]. Importantly, the improvement of mitochondrial function and the antagonism of glutamate neurotoxicity of hippocampal neurons via activating Shh signaling pathway had been reported [21, 22], which indicated that Shh/Ptch1-mediated mitochondrial function and glutamate neurotoxicity played a critical role in alleviating damage of chronic hypoperfusion in MID.

*Kaixin San* (KXS), a classic prescription of *Beiji Qianjin Yaofang*, was used to treat “amnesia” for thousand years in China and Southeast Asia. KXS is composed of *Panax ginseng* C.A. Meyer. (Renshen), *Poria cocos* (Schw.) Wolf (Fuling), *Polygala tenuifolia* Willd. (Yuanzhi), and *Acorus tatarinoxjuii* Schott (Shichangpu). KXS had been reported to improve cognitive dysfunction in many animal models of Alzheimer's disease (AD). Increasing number of studies had also shown that these four herbs or combinations of them were frequently used to alleviate the cognitive deficit in dementia, such as VD and AD [23–25]. Our previous studies had shown that KXS could alleviate memory impairment through decreasing the content of *γ*-aminobutyric acid (GABA) and inducible nitric oxide synthase (iNOS) or elevating the ATP content of brain tissue in MID rats [26]. However, the underlying mechanisms of KXS treating MID remain unclear.

In this study, we investigated the effects of KXS on rescuing cognitive damage in MID rats based on its effects on regulating mitochondrial function and glutamate neurotoxicity via activating the Shh/Ptch1 signaling pathway. Our data showed that KXS provided a neuroprotective effect on cognitive impairment via improving mitochondrial function and inhibiting glutamate neurotoxicity by activating Shh/Ptch1 signaling pathway in MID rats. Furthermore, our data also proved that Shh/Ptch1 signaling pathway played a key role of against glutamate neurotoxicity of KXS in vitro.

## 2. Materials and Methods

### 2.1. Animals and Cell Line

65 male Sprague-Dawley rats (200 ± 20 g, SPF) were purchased from Chengdu dashuo experimental animal Co., Ltd. (Chengdu, China) and used in this research. All rats were housed in an animal observation room of Chengdu University of Traditional Chinese Medicine (temperature: 22 ± 2°C, humidity: 65 ± 5%, 12-hour light/dark cycle) and were free to access water and food. The experimental procedure was approved by the Ethics Committee for Animal Experiments of the Institute of Material Medica Integration and Transformation for Brain Disorders (Chengdu University traditional Chinese medicine, No. IBD2017013) and carried out in accordance with the NIH Guide for the Care and Use of Laboratory Animals (NIH Publication No. 85-23, 1985, revised 1996). Rat pheochromocytoma-derived cell line PC12 cells (high differentiation) were obtained from the cell bank of the Chinese Academy of Sciences (Shanghai, China).

### 2.2. Materials and Reagents

Mesylate dihydroergotoxine tablets (Lot No. 7C883T) were purchased from Tianjin Huajin Pharmaceutical Co., Ltd. (Tianjin, China). Adenosine triphosphate (ATP) disodium salt (Lot No. 1001665443), adenosine diphosphate (ADP) disodium salt (Lot No. 101606467), adenosine monophosphate (AMP) disodium salt (Lot No. 1001726877), and glutamate (Lot # SLCD3953) were obtained from Sigma-Aldrich (USA). Cyclopamine (Cyc, Cat.# HY-17024/CS-0633) was provided by Med Chem Express (MCE). The glutamic acid measurement kit (Cat.NoA07411) was supplied by Nanjing Jiancheng Bioengineering Institute (Nanjing, China). Tissue mitochondria isolation kit (Cat.NoC3606) was furnished by Beyotime Institute of Biotechnology (Shanghai, China). JC-1 kit (Cat. No 551302) was produced by Becton-Dickinson and Company (USA). Primary antibodies, including Smo (66851-1-Ig) and Gli1 (66905-1-Ig), were provided by Wuhan Sanying Biotechnology Co., Ltd., (Wuhan, China); NMDAR1 (5704S) and p-NMDAR1 (3384S) were obtained from Cell Signaling Technology (USA); Ptch1 (NBP1-71662) was provided by Novus (USA).

### 2.3. Preparation and Analysis of KXS Extract

*Panax ginseng* C.A. Meyer. (Renshen), *Poria cocos* (Schw.) Wolf (Fuling), *Polygala tenuifolia* Willd. (Yuanzhi), and *Acorus tatarinoxjuii* Schott (Shichangpu) were purchased from the pharmacy of Affiliated Hospital of Chengdu University of Traditional Chinese Medicine (Chengdu, China). Renshen, Fuling, Yuanzhi, and Shichangpu were mixed at a ratio of 3 : 3 : 2 : 2 as reported previously [23, 27]. The above four Chinese herbal pieces were soaked with 6 times of distilled water for 1 hour and then boiled 3 times for 1.5 h every time. The three decoctions were merged, filtered, and condensed to 0.6 g/mL at 80°C for animals administrated or 10 g/mL for high-performance liquid chromatography (HPLC) analysis. A moderate amount of KXS extracts was dissolved in methanol, centrifuged (13,000 rpm, 10 min), and filtered through 0.22 *μ*m filter before HPLC analysis. The Agilent 1260 Infinity High-Performance Liquid Chromatography instrument (Agilent Technologies, Inc.) was used to analyze the KXS extract solution, and chromatographic separation was performed using an Agilent C18 Column (4.6 × 250 mm, 5 *μ*m; Agilent Technologies, Inc.) with a column temperature of 25°C, flow rate of 1 mL/min, and injection volume of 10 *μ*L. A gradient elution was provided with 0.1% formic acid water as solvent A (80%~20%) and acetonitrile as solvent B (20%~80%), and the effluent absorbance was measured at 210 nm and 320 nm. The concentration of ginsenoside Rg1, ginsenoside Rd, ginsenoside Rb1, polygalaxanthone III, and 3,6′-disinapoyl sucrose were used as reference compounds to verify the main components of KXS extracts. For cell studies, the extracts were dried by a freeze dryer into powder for storage. Before the treatment, the powder was redissolved with water and filtered through a 0.22 *μ*m filter to obtain KXS extracts.

### 2.4. Animal Model and Administration

As previously reported, an animal model of MID was established by injecting homologous blood emboli into the right internal carotid artery of male Sprague-Dawley rats [28]. Briefly, rat microthrombus of 150-180 *μ*m fine particles was prepared in 20 mg/6 mL normal saline suspension early. After rats were anesthetized by pentobarbital sodium 40 mg/kg, the right internal carotid artery was exposed, and 0.4mL microthrombus suspension was injected into the right internal carotid artery in each rat to induce MID. The same surgical procedure was implemented in the sham-operation group, but microthrombus suspension was replaced with 0.4 mL normal saline. Our previous study had proved that the dose (high-dose KXS, 2.12 g/kg and low-dose KXS, 1.06 g/kg) and the duration of treatment (once a day for 45 days) of KXS had great therapeutic effects on MID rats [26]. Therefore, we refer to the previous dose and time of administration of KXS in this experiment. Two weeks later, the survive rats (*n* = 50) were randomly divided into model group (*n* = 10), hydergine group (0.7 mg/kg, *n* = 10, hydergine is the most commonly used vasodilator in the treatment of MID, we used it as a positive control to evaluate rats model and effectiveness of KXS), high-dose KXS group (KXS H, 2.12 g/kg, *n* = 10), and low-dose KXS group (KXS L, 1.06 g/kg, *n* = 10) according to body weight. Besides, 10 sham-operated rats were used as control group. All animals were orally 10 mL/kg administered with respective drugs except the control group (administered with normal saline) once a day for 45 d.

### 2.5. Morris Water Maze Test

Eight rats in each group were tested in Morris Water Maze to assess the cognitive function. The rats were placed in an EthoVision XT Morris water maze video tracking test system that can automatically record time for rats to find the platform. The procedure was performed as previously described [29]. On the 40th day of drug intervention, a 5-day hidden platform experiment started. The training time was 60 s, and all animals were trained twice a day. The time of finding the platform was recorded as the escape latency. Attentively, it needed to be artificially guided to stay on the platform for 10 s, and the escape latency was recorded as 60 s if the rat did not find the platform in the first five days. On the sixth day, the platform was removed to test the spatial search ability of the rats, following the frequency of rats passed through where the platform was initially placed was recorded [30], the percent of time staying in target quadrant and the time of first arrival at the platform zone within 60 s were calculated.

### 2.6. Hematoxylin and Eosin Staining

The left hemisphere of six rats in each group was fixed with 4% paraformaldehyde, dehydrated, and embedded, cut into 5 *μ*m slices, then stained by hematoxylin for 30 min and eosin for 5 min, vitrified with xylene, and sealed with neutral resin. The stained slices were observed and photographed under an optical microscope for examining the pathological changes in the hippocampal CA1 region.

### 2.7. Measurement of the Mitochondrial Swelling and MMP

Mitochondrial swelling and mitochondrial membrane potential (MMP) were measured with flow cytometry (FCM). The hippocampal mitochondria of three rats in each group were extracted strictly according to the tissue mitochondria isolation kit. The isolated mitochondria were resuspended with mitochondria storage buffer and adjusted to a concentration of 1 × 10^5^ cells/mL per tube, then detected with flow cytometry (Bio-Rad, ZE5) for mitochondrial swelling. The ratio of FSC/SSC reflects the degree of mitochondrial swelling. Similarly, hippocampal cell suspension of 1 × 10^5^ cells/mL was prepared, centrifuged, and resuspended with 500 *μ*L of JC-1 working solution. Then incubated at 37°C for 15 min, resuspended cells with PBS, then analyzed by flow cytometry for MMP. The ratio of red/green (PE /FITC) fluorescence intensity reflects the level of MMP.

### 2.8. Detection of Hippocampus Energy Charge (EC) with HPLC

A total of 70 mg of hippocampus tissue was collected, mixed with normal saline (weight of hippocampus : normal saline volume = 1 : 10), and then disrupted with a homogenizer. Then, 0.6 mL homogenate was needed, add an equal volume of 0.5 mol/L perchloric acid, and centrifuged for 10 min at 12000g at 4°C. Furthermore, 0.8 mL supernatant was collected, add 45 *μ*L NaOH (5 mol/L) to neutralize PH, and centrifuged for 10 min at 12000 g at 4°C after standing for 30 min. The supernatant was used for subsequent HPLC detection. The Agilent 1260 Infinity High-Performance Liquid Chromatography instrument (Agilent Technologies, Inc.) was used to detect the contents of AMP, ADP, and ATP, and chromatographic separation was performed using an Agilent C18 Column (4.6 × 250 mm, 5 *μ*m; Agilent Technologies, Inc.) with a column temperature of 25°C, flow rate of 1 mL/min, and injection volume of 10 *μ*L. A gradient elution was provided with 0.05% phosphate buffer (PH = 6.5) as solvent A (98%~90%) and methanol as solvent B (2%~10%), and the effluent absorbance was measured at 254 nm. The energy charge (EC) was calculated by the following formula: EC = ([ATP] + 1/2[ADP])/([ATP] + [ADP] + [AMP]).

### 2.9. Enzyme-Linked Immunosorbent Assay

A total of 60 mg of brain tissue was weighed and mixed with PBS containing 1% PMSF. Then, the brain tissue was grounded with a homogenizer and then centrifuged for 10 min at 5000 g at 4°C. The supernatant was collected. Subsequently, supernatant (100 *μ*L/well) was used to determine the concentrations of glutamate strictly according to the kit instructions.

### 2.10. Western Blot Analysis

An appropriate amount of brain tissue was lysed in RIPA lysate containing 1% PMSF, 1% phosphatase inhibitor, and centrifuged at 4°C, 12000 g for 10 min. The supernatant was collected for detecting protein concentration and western blot analysis. The target protein was separated with SDS-PAGE, transferred to a PVDF membrane, sealed with 5% BSA at room temperature for 90 min, and incubated with the primary antibodies at 4°C overnight: anti-Ptch1 (1 : 1000), anti-Smo (1 : 1000), anti-Gli1 (1 : 1000), anti-NMDAR1 (1 : 1000), anti-p-NMDAR1 (1 : 1000), and anti-GAPDH (1 : 5000). Subsequently, the target protein was washed with TBST solution three times, incubated with the secondary antibody (1 : 5000) at room temperature for 90 min, washed with TBST solution three times again, and visualized by hypersensitive ECL kit. The relative expression of the target proteins was analyzed using the Quantity One software (Bio-Rad, USA).

### 2.11. Cell Culture and Cell Viability Assay

PC12 cells were maintained in DMEM containing 10% fetal bovine serum (FBS), 1% Penicillin streptomycin in a 5% CO_2_ incubator at 37°C. PC12 cells were employed to investigate the effect of KXS on antiglutamate neurotoxicity. To evaluate the effect of glutamate on the PC12 cell viabilities, cells were presented with glutamate at concentrations of 2.5, 5, 10, 20, and 40 mM for 24 h (37°C, 5% CO_2_). To evaluate the effect of KXS and hydergine on cell viability, KXS at the final concentrations of 31.25, 62.5, 125, 250, and 500 *μ*g/mL and hydergine at the final concentrations of 15.625, 31.25, 62.5, and 125 *μ*g/mL were added and cultured (37°C, 5% CO_2_) for 24 h. In addition, to evaluate the key role of KXS in antiglutamate neurotoxicity via Shh/Ptch1 signaling pathway, PC12 cells were divided into control, model (glutamate, 5 mM), Glu+hydergine (100 *μ*g/mL), Glu+KXS L (freeze-dried powder, 50 *μ*g/mL), Glu+KXS H (freeze-dried powder, 100 *μ*g/mL), and Glu+KXS H+cyclopamine (100 *μ*g/mL, 5 *μ*M). The cells of Glu+hydergine, Glu+KXS L, Glu+KXS H, and Glu+KXS H+cyclopamine groups were pretreated with the corresponding concentration of drugs and were incubated for 2 h before stimulation with glutamate (5 mM) for 24 h. The cells of the model group were only stimulated with 5 mM glutamate for 24 h. Afterward, 10 *μ*L of MTT (5 mg/mL) was added to each well and incubated at 37°C for 4 h. The formazan crystals were solubilized in 100 *μ*L of dimethylsulfoxide (DMSO), and the absorbance was measured at 540 nm using a microplate reader (Thermo Labsystems, Franklin, MA, USA). Cell viability was calculated as a percentage of the vehicle control group [31]. All of the absorption values were calculated by averaging the results of each sample in triplicate.

### 2.12. Statistical Analysis

Data were expressed as mean ± standard error of the mean (SEM). Morris water maze escape latency data were analyzed with a two-way analysis of variance (ANOVA) with Tukey's multiple comparisons test. The other data were analyzed by one-way ANOVA followed by Dunnett's multiple comparison test. The significance of statistical differences was considered at *p* < 0.05.

## 3. Results

### 3.1. Quality Control of KXS Extracts

**Figure 1:**
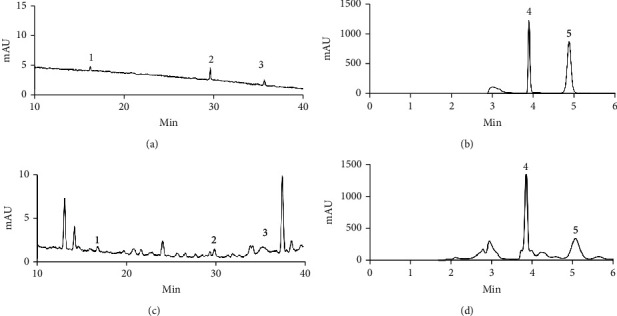
The main components of KXS extract were determined by HPLC. (a) Renshen standards liquid. (b) Yuanzhi standards liquid. (c and d) The KXS extracts. Peak 1: ginsenoside Rg1; peak 2: ginsenoside Rb1; peak 3: ginsenoside Rd; peak 4: polygalaxanthone III; peak 5: 3,6′-disinapoyl sucrose.

To control the quality of KXS extracts, we used HPLC to quantify the main components. A typical HPLC profile was developed for KXS extracts (Figure 1), which served as an index for the identification of KXS. Ginsenoside Rg1, ginsenoside Rb1, ginsenoside Rd, polygalaxanthone III, and 3,6′-disinapoyl sucrose are characteristic components of KXS and were detected by HPLC. The contents of those signature ingredients were 0.444 ± 0.003 *μ*g/mg, 0.330 ± 0.001 *μ*g/mg, 0.877 ± 0.001 *μ*g/mg, 0.745 ± 0.0003 *μ*g/mg, and 1.142 ± 0.0006 *μ*g/mg, respectively.

### 3.2. KXS Ameliorated Cognitive Impairment and Hippocampal CA1 Neuron Damage in MID Rats

**Figure 2:**
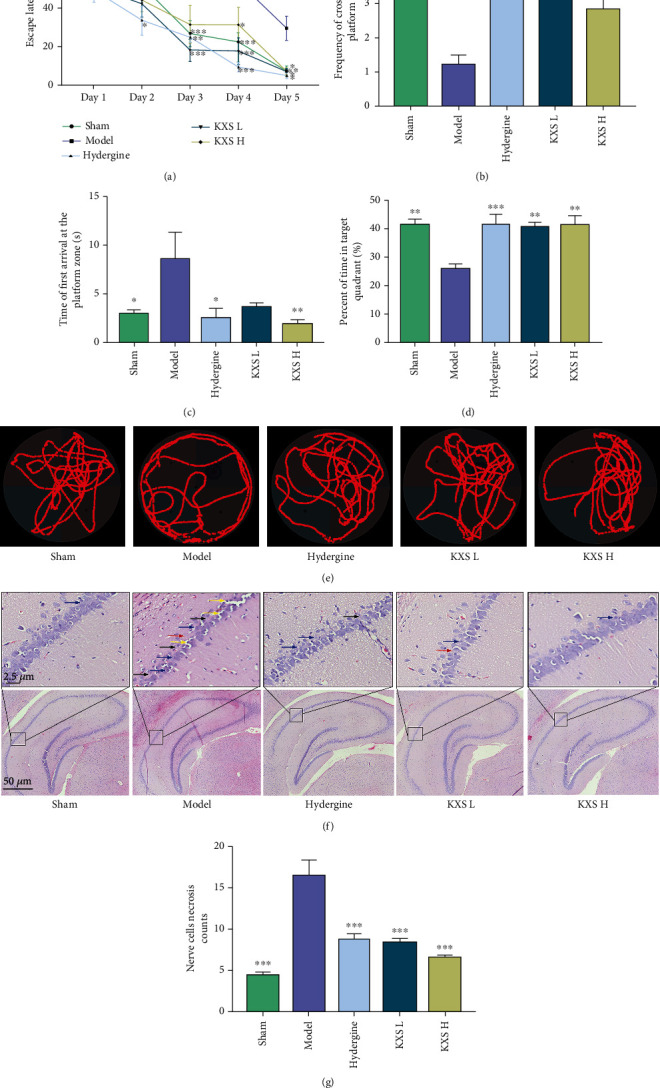
KXS alleviated cognitive impairment and hippocampal CA1 neuronal damage in MID rats. Escape latency (a, *n* = 8), frequency of crossing the platform (b, *n* = 8), time to first reach the platform zone (c, *n* = 8), percent of time staying in the target quadrant (%, d, *n* = 8), representative traces of each group (e) in Morris Water Maze test. (f) Representative pictures of hematoxylin and eosin staining showed KXS extracts attenuated the damage to the hippocampus CA1 region in the MID rats (magnification: 200×). Nerve cell necrosis and cell degeneration (blue arrow), a decrease in the number of neurons (red arrow) and an enlarged gap (black arrow), the darkening of the nuclei (yellow arrow). (g) The necrotic cells count in the CA1 region (*n* = 6). The above data were presented as mean ± SEM. ^∗^*p* < 0.05, ^∗∗^*p* < 0.01, and ^∗∗∗^*p* < 0.001 compared with the model group. The escape latency was performed by two-way ANOVA with Tukey's multiple comparisons test, and the others were performed by one-way ANOVA with Dunnett's multiple comparison test.

To evaluate whether KXS could ameliorate the memory dysfunction of MID rats, we conducted the Morris Water Maze test for these rats. As Figure 2(a) shows, the escape latency of each group (except for the model group) remarkably shortened constantly with the increased training days, and the model group exhibited longer escape latency than the sham group (*F*(1, 10) = 30.96, *p* < 0.001); administration of KXS-L (*F*(1, 10) = 33.9, *p* < 0.001) and KXS-H (*F*(1, 10) = 19.27, *p* < 0.01) significantly attenuated the increased latency to reach the platform. The results of the spatial search test are shown in Figures 2(b)–2(d), the frequency of crossing the platform and the percent of time staying in the target quadrant were decreased in the model group compared with the sham group (*p* < 0.05 or *p* < 0.01). The MID rats treated with KXS-L and KXS-H both increased the time staying in the target quadrant (*p* < 0.01), and KXS-L can also increase the frequency of crossing the platform of MID rats (*p* < 0.01). Besides, MID rats spent more time in arriving at the platform zone (*p* < 0.05), which can be shorten by hydergine and high-dose KXS (*p* < 0.05 or *p* < 0.01). The above results showed that KXS could ameliorate the memory impairment of MID rats.

The hippocampal CA1 region is very sensitive to ischemic injury and closely related to cognitive function [32]. To further investigate the role of KXS in alleviating cognitive dysfunction, we detected the hippocampus pathology of MID rats. As Figure 2(f) shows, compared with the sham group, the pathology changes of hippocampus CA1 region such as nerve cells necrosis and cell degeneration (blue arrow), a decrease in the number of neurons (red arrow) and an enlarged gap (black arrow), the darkening of the nuclei (yellow arrow), were obvious in the model group while those pathological characteristics were markedly alleviated after treatment with KXS and hydergine. Subsequently, we counted the number of necrotic cells in the hippocampus CA1 region (Figure 2(g)). Our results showed that the number of necrotic cells in the model group was significantly increased compared with the sham group (*p* < 0.001). In the rats treated with hydergine or KXS, the number of necrotic cells was significantly reduced (*p* < 0.001). These results suggested KXS could protect against hippocampal histopathological alterations in MID rats.

### 3.3. Effects of KXS on Hippocampal Mitochondrial Functions in MID Rats

**Figure 3:**
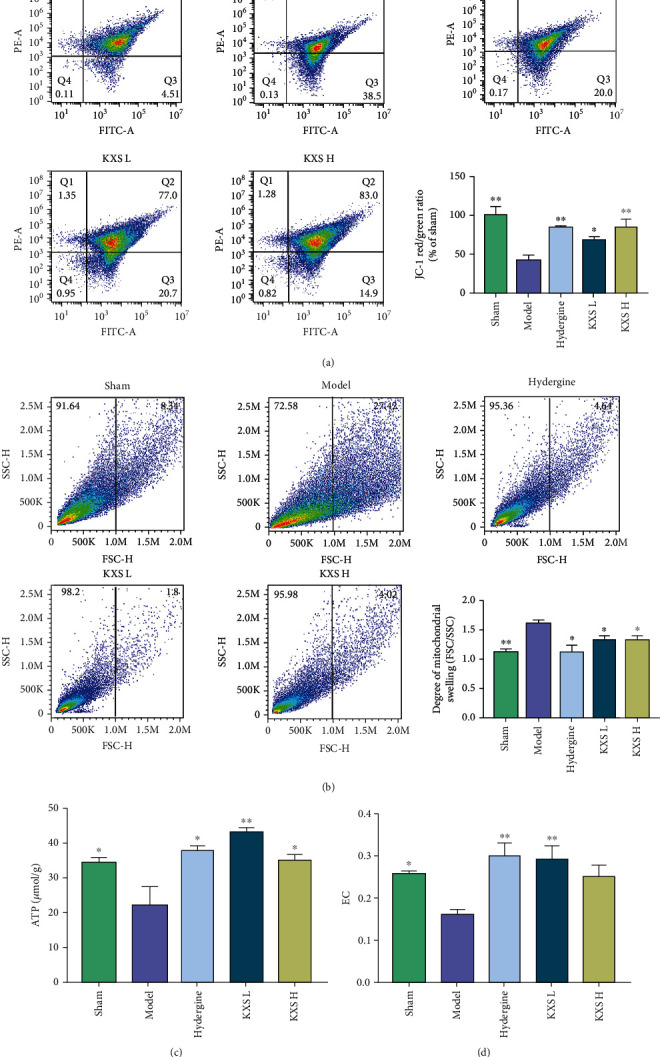
Effects of KXS on hippocampal mitochondrial functions in MID rats. (a) Flow cytometry analysis and quantification of mitochondrial membrane potential (MMP). (b) Flow cytometry analysis and quantification of mitrochondrial swelling. (c) The content of ATP in MID rats. (d) The level of EC. The above data were presented as mean ± SEM (*n* = 3). ^∗^*p* < 0.05, ^∗∗^*p* < 0.01, and ^∗∗∗^*p* < 0.001 compared with the model group. Comparisons were performed by one-way ANOVA with Dunnett's multiple comparison test.

Hippocampal mitochondrial dysfunction plays a major role in the pathogenesis of VD [8, 11]. MMP and mitochondrial swelling are considered important parameters assessing mitochondrial bioenergy. To further investigate the role of KXS on mitochondrial functions, we detected MMP and mitochondrial swelling in the hippocampus of MID rats. As Figure 3(a) shows, MMP decreased significantly in the model group compared with the sham group (*p* < 0.01). However, the above lesion was reversed both in KXS-L and KXS-H groups (*p* < 0.05 or *p* < 0.01). As Figure 3(b) shows, mitochondrial swelling was increased in the model group compared with the sham group (*p* < 0.01), while the pathological changes were ameliorated both in KXS-L and KXS-H groups (*p* < 0.05).

Mitochondria supply the necessary energy to support the survival and function of neurons. As Figures 3(c) and 3(d) shows, the ATP and EC were decreased in the model group (*p* < 0.05); however, the content of ATP was significantly increased in the KXS-L and KXS-H groups compared with the model group (*p* < 0.01 or *p* < 0.05). Besides, the brain EC was elevated in the KXS-L group (*p* < 0.01). Given the findings mentioned above, we believed KXS has protective effects on hippocampal mitochondria.

### 3.4. Effects of KXS on Glutamate Neurotoxicity

**Figure 4:**
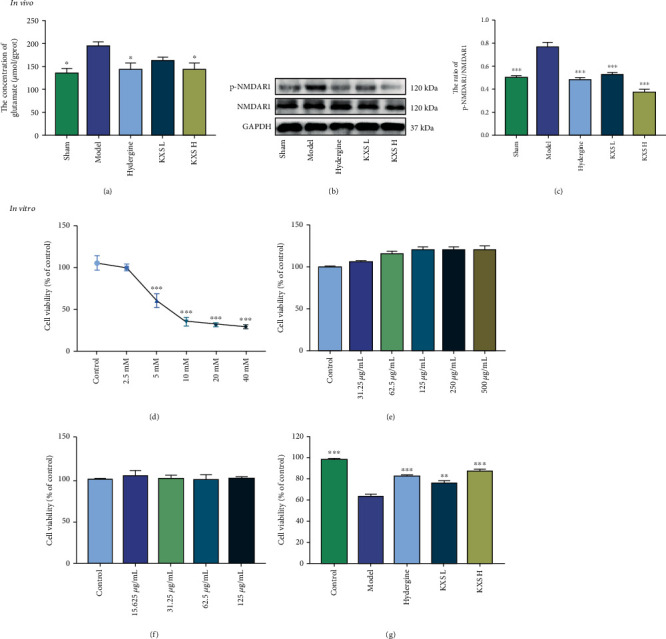
Effects of KXS on the concentration of glutamate and expression of p-NMDAR1 in MID rats and anti-glutamate neurotoxicity in vitro. (a) Concentration of glutamate in MID rats brain tissue (mean ± SEM, *n* = 5). (b) Representative western blot images of p-NMDAR1 and NMDAR1. (c) Quantification of p-NMDAR1/NMDAR1 ratio (mean ± SEM, *n* = 3). (d) The viability of the different doses of glutamate in PC12 cells (mean ± SEM, *n* = 3). (e) The viability of the different doses of KXS in PC12 cells (mean ± SEM, *n* = 3). (f) The viability of the different doses of hydergine in PC12 cells (mean ± SEM, *n* = 3). ^∗^*p* < 0.05, ^∗∗^*p* < 0.01, and ^∗∗∗^*p* < 0.001 compared with the model group. Comparisons were performed by one-way ANOVA with Dunnett's multiple comparison test.

Excessive extracellular glutamate can lead to the overactivation of postsynaptic glutamate receptors, like N-methyl-D-aspartate receptor 1 (NMDAR1), which evokes mitochondrial dysfunction and neuronal dysfunction [33, 34]. Considering the above factors, we wonder if KXS's protective effects on mitochondria and neurons are associated with reducing the content of glutamate and p-NMDAR1. As Figures 4(a)–4(c) shows, the concentration of glutamate and expression level of p-NMDAR1 were both increased in the model group compared with the sham group (*p* < 0.05 or *p* < 0.001). On the contrary, the concentration of glutamate was reduced in KXS H (*p* < 0.05), and the expression level of p-NMDAR1 was significantly downregulated in both KXS L and KXS H groups (*p* < 0.001) compared with the model group. This experiment showed KXS could decrease the concentration of glutamate and the level of p-NMDAR1 in MID rats.

To further study the against glutamate neurotoxicity of KXS, we used glutamate to induce neurotoxicity in PC12 cells and used this model to evaluate the antiglutamate neurotoxicity of KXS. The results of glutamate, KXS, and hydergine on the cell viabilities of PC12 cells were presented in Figures 4(d)–4(f). It could be found that KXS did not show any toxicity of up to the concentration of 500 *μ*g/mL, and hydergine did not show any toxicity of up to the concentration of 125 *μ*g/mL. However, glutamate at concentrations of 2.5-40 mM has significant toxicity on PC12 cells under 24 h culture time, and the cell viability of PC12 cells was reduced to 60.71% after treated with 5 mM glutamate for 24 h. Thus, glutamate at the concentrations of 5 mM for 24 h was used to establish neurotoxicity. As shown in Figure 4(g), the cell viability was significantly increased by treatment with KXS and hydergine compared with the model group (*p* < 0.01 or *p* < 0.001). Consequently, these results indicated that KXS can effectively reduce the concentration of glutamate and level of p-NMDAR1 in MID rats and effectively inhibit the neurotoxicity induced by glutamate in PC12 cells.

### 3.5. KXS Activated Shh/Ptch1 Pathway in MID Rats and against Glutamate Neurotoxicity in PC12 Cells via Shh/Ptch1 Pathway

**Figure 5:**
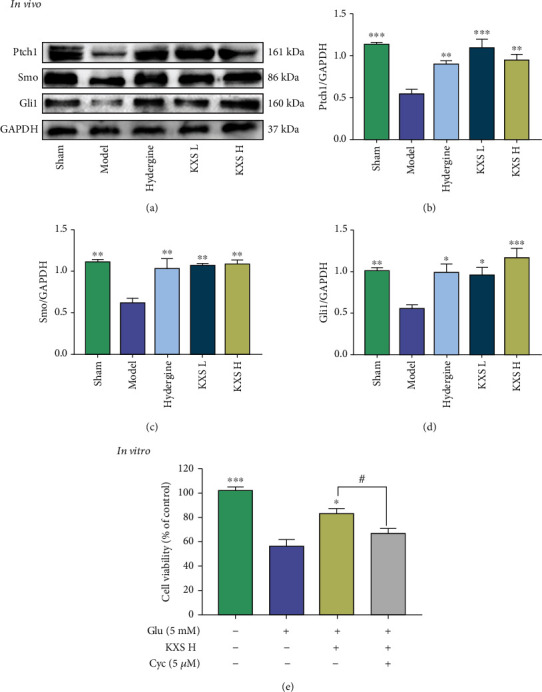
KXS activated Shh/Ptch1 pathway in MID rats and against glutamate neurotoxicity in PC12 cells via Shh/Ptch1 pathway. (a) Representative western blot images of Ptch1, Smo, and Gli1. Quantifications of Ptch1/GAPDH (b), Smo/GAPDH (c), and Gli1/GAPDH ratio (d). (e) The effect of KXS H and cyclopamine on glutamate-induced PC12 cells neurotoxicity. Above data were presented as mean ± SEM (*n* = 3). ^∗^*p* < 0.05, ^∗∗^*p* < 0.01, and ^∗∗∗^*p* < 0.001 compared with the model group. ^#^*p* < 0.05 compared with the Glu+KXS H group. Comparisons were performed by one-way ANOVA with Dunnett's multiple comparison test.

Recently, studies have shown that activating the Shh signaling pathway can increase mitochondrial activities and protect hippocampal neurons against glutamate-induced neurotoxicity [21]. To illuminate whether the effects of KXS on improving mitochondrial function and reducing glutamate neurotoxicity were related to Shh/Ptch1 signaling pathway, we detected the protein expression of Ptch1, Smo, and Gli1. As Figures 5(a)–5(d) show, the protein levels of Ptch1, Smo, and Gli1 were significantly decreased (*p* < 0.01 or *p* < 0.001) in the model group compared with the sham group. After treatment with KXS, the protein levels of Ptch1, Smo, and Gli1 were upregulated, respectively (*p* < 0.05, *p* < 0.01, or *p* < 0.001). To evaluate the key role of Shh/Ptch1 signaling pathway in antiglutamate neurotoxicity of KXS, we detected the cellular activity of PC12 under glutamate stimulation conditions. As Figure 5(e) shows, cyclopamine (a Shh pathway antagonist) could significantly inhibit the protective effect of KXS on glutamate neurotoxicity in PC12 cells (*p* < 0.05).

## 4. Discussion

*Kaixin San* is a classic Chinese medicine prescription for forgetfulness and has been testified to improve cognitive impairment in many types of animal dementia models [23, 24, 35, 36]. Our present study demonstrates that treatment with KXS (i) improves cognitive dysfunction and alleviates hippocampal neuron damage of MID rats, (ii) rescues mitochondrial dysfunction through upregulating brain energy, alleviating mitrochondrial swelling and improving MMP under chronic hypoperfusion conditions, (iii) inhibits glutamate neurotoxicity via decreasing the content of glutamate and the level of p-NMDAR1 in MID rats, and (iv) activates Shh/Ptch1 signaling pathway in MID rats and protects PC12 cells against glutamate neurotoxicity through Shh/Ptch1 signaling pathway. Collectively, improving mitochondrial quality and inhibiting glutamate neurotoxicity via activating Shh/Ptch1 signaling pathway constituted part of an essential mechanism of KXS to provide the neuroprotective effect on MID rats.

Multiple-infarct dementia (MID) is the most common type of vascular dementia (VD). MID's clinically pathological changes are common in atherosclerosis and hypertensive arteriole disease [37]. The thrombus fragments fall off and enter the blood circulation after arteriosclerosis. When thrombus fragments pass the small branches of the cerebral blood vessels form blockages, which results in brain tissue ischemia and hypoxia (chronic hypoperfusion) and then causes brain tissue damages and cognitive impairment [3, 38]. So chronic hypoperfusion is considered to contribute significantly to brain tissue damage and cognitive decline. Multiple infarcts and thromboembolism model are the most relevant model to VD [38]. In this study, the microthromboembolism-induced MID rats were used to simulate conditions of chronic cerebral hypoperfusion and utilized to evaluate the effects of KXS on cognitive dysfunction. As a vasodilator, clinical research had shown that hydergine could increase oxygen uptake as well as improve cerebral blood flow and decrease vascular resistance in patients with cerebrovascular disease, such as VD and MID [39]. So it is commonly used for treating patients with either dementia or “age-related” cognitive symptoms [40]. Like previous studies [41–43], we used hydergine as a positive control as well. Our results showed that the learning-memory ability was severely impaired in about 2 months after microthromboembolism surgery in rats, and these results were consistent with previous studies in MID model rats [4, 28]. Moreover, our data also showed that KXS could ameliorate the cognitive impairment of MID. It is widely known that the hippocampus CA1 region is an important area that is associated with learning and memory impairment [44]. Similar to the above results, the results of our study illuminated that nerve cell necrosis and cell degeneration were obvious in the model group rats while these changes dramatically reduced after KXS treatment. These results showed KXS alleviated cognitive impairment and hippocampal neuron injury in MID rats.

Glutamate plays an important role in neuronal development, axon guidance, brain development and maturation, learning and memory, and synaptic plasticity [45, 46]. But, excessive concentration of extracellular glutamate is neurotoxicity, known as glutamate excitotoxicity, which is the leading mechanism of neuronal death and cognitive disorder in cerebral ischemia disease [9, 47, 48]. It is reported that hypoperfusion (a main contributor to MID) can trigger hypoxia and glucose deprivation in nerve cells, which results in membrane depolarization and glutamate outflow [49]. Studies have reported that glutamate acts on glutamate receptors, such as the N-methyl-D-aspartate receptors (NMDARs), specifically the NMDAR1 subunit, and leads to neuronal hyperexcitability and death [50, 51]. Consistent with previous studies, our study showed that MID-induced chronic cerebral hypoperfusion increased the content of glutamate and p-NMDAR1, while KXS reduced the levels of glutamate and p-NMDAR1. In addition, our results also showed KXS could effectively inhibit the neurotoxicity of glutamate in glutamate-induced PC12 cells. Therefore, it is reasonable to suspect that antiglutamate neurotoxicity plays an important role in the neuroprotective effect of KXS.

Hippocampus is key for spatial learning and memory, which is most sensitive to ischemic insult, and its mitochondrial damage plays an important role in the pathogenesis of VD [8]. Excessive glutamate can also induce mitochondrial dysfunction through activating NMDA receptors, which results in Ca^2+^ influx and MMP collapse [34]. Glutamate excitotoxicity can induce mitochondrial dysfunction, and mitochondrial damage can exacerbate glutamate excitotoxicity as well. For instance, energy failure, an early consequence of hypoxia-ischemia and the key sign of mitochondrial dysfunction, renders neurons vulnerable to excitotoxicity [52]. Mitochondrial energy metabolism, MMP, and mitochondrial swelling are major parameters to assess mitochondrial bioenergy [53, 54]. In our study, KXS increased ATP content and hippocampus MMP and decreased the degree of mitochondrial swelling in the MID rats, which revealed that KXS can improve mitochondrial quality and against glutamate excitotoxicity to ameliorate learning and memory impairment and neuron damage in MID rats.

Shh/Ptch1 signaling pathway is involved in cell development, survival, and differentiation in kinds of cells, including neurons [55]. Dysfunction or aberrant activation of the Shh signaling pathway is associated with developmental deformities and cancers [56]. Recently, beneficial actions of activating canonical Shh/Ptch1 signaling pathway in cerebral ischemic injury have been reported, such as enhancing neurogenesis and white matter remodeling, antioxidation, antiexcitotoxicity, and antiapoptosis [18, 20, 57, 58]. Besides, a study reported the effects of activating the Shh/Ptch1 signaling pathway on reducing glutamate neurotoxicity by “nNOS-Sox2-Shh” axis, which functions as a novel feedback compensatory mechanism to protect neurons against the early excitotoxicity and ischemic injury [59]. Furthermore, an increasing number of studies had shown that Shh/Ptch1 signaling pathway can also protect neurons against pathological stressors that cause or promote neuronal dysfunction by increasing mitochondrial mass and function [18, 21]. The Shh/Ptch1 signaling pathway mainly consists of Shh ligand, patched (Ptch1), and Smoothened (Smo) receptors, and Gli transcription factors (Gli1, Gli2, and Gli3). When the Shh ligand binds to Ptch1, it relieves the repression on Smo and ultimately activates Gli transcription factors [60]. Gli transcription factors, especially Gli1, are used as indicators of the activation of the Shh signaling pathway [61, 62]. In this experiment, we demonstrated that KXS could activate Shh/Ptch1 signaling pathway with upregulating the expression levels of Ptch1, Smo, and Gli1 in MID rats (Figures 5(a)–5(d)). Furthermore, cellular level experiment indicated that the inhibition of the Shh/Ptch1 signaling pathway by cyclopamine can inhibit the protective effect of KXS on glutamate-induced neurotoxicity in PC12 cells (Figure 5(e)). The above results suggested the neuroprotective effects of KXS (improved mitochondrial quality and inhibited glutamate neurotoxicity) in MID rats may work through activating the Shh/Ptch1 signaling pathway.

## 5. Conclusion

**Figure 6:**
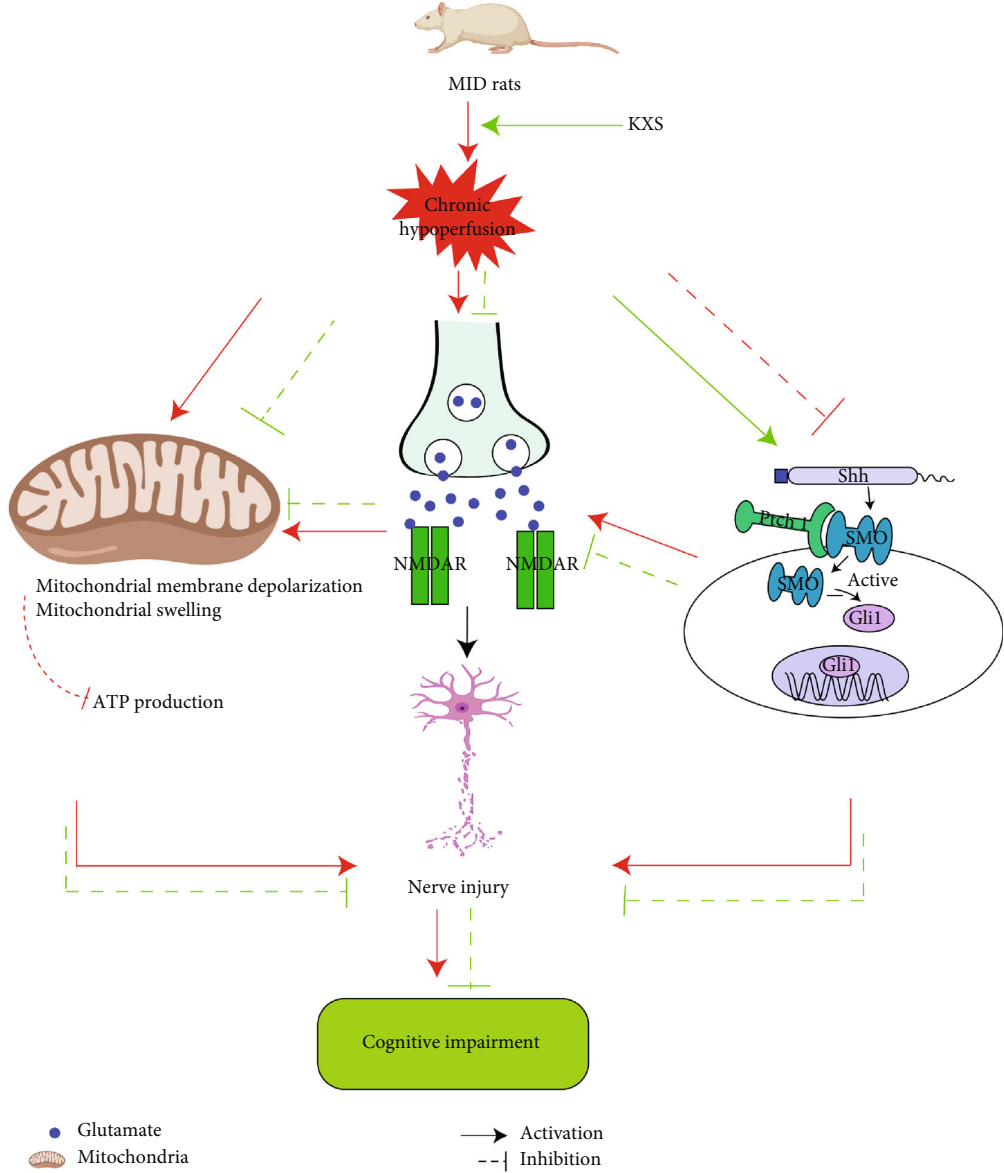
Schematic of the molecular mechanism of KXS protecting rats against MID via Shh/Ptch1 signaling pathway.

In summary, this study is the first to demonstrate that KXS could resist glutamate neurotoxicity and rescue mitochondrial function through activating the Shh/Ptch1 signaling pathway, thereby easing cognitive dysfunction and neurological deficits of MID (Figure 6). The present study may further offer basic information about the neuroprotective effect of KXS and indicate that KXS might be developed as an effective intervention drug of MID.

## Data Availability

The data used to support the findings of this study are available from the corresponding author upon request.
